# Poverty and neighborhood opportunity effects on neonate DNAm developmental age

**DOI:** 10.1371/journal.pone.0306452

**Published:** 2024-07-12

**Authors:** Stefanie R. Pilkay, Anna K. Knight, Nicole R. Bush, Kaja LeWinn, Robert L. Davis, Frances Tylavsky, Alicia K. Smith

**Affiliations:** 1 School of Social Work, Syracuse University, Syracuse, New York, United States of America; 2 Department of Gynecology and Obstetrics, Emory University School of Medicine, Atlanta Georgia, United States of America; 3 Department of Psychiatry, University of California San Francisco School of Medicine, San Francisco, San Francisco, California, United States of America; 4 Department of Pediatrics, University of California San Francisco, San Francisco, San Francisco, California, United States of America; 5 Health and Science Center, University of Tennessee, Memphis, Tennessee, United States of America; University of Lausanne: Universite de Lausanne, SWITZERLAND

## Abstract

**Background:**

Children from families with low socioeconomic status (SES), as determined by income, experience several negative outcomes, such as higher rates of newborn mortality and behavioral issues. Moreover, associations between DNA methylation and low income or poverty status are evident beginning at birth, suggesting prenatal influences on offspring development. Recent evidence suggests neighborhood opportunities may protect against some of the health consequences of living in low income households. The goal of this study was to assess whether neighborhood opportunities moderate associations between household income (HI) and neonate developmental maturity as measured with DNA methylation.

**Methods:**

Umbilical cord blood DNA methylation data was available in 198 mother-neonate pairs from the larger CANDLE cohort. Gestational age acceleration was calculated using an epigenetic clock designed for neonates. Prenatal HI and neighborhood opportunities measured with the Childhood Opportunity Index (COI) were regressed on gestational age acceleration controlling for sex, race, and cellular composition.

**Results:**

Higher HI was associated with higher gestational age acceleration (*B* = .145, *t* = 4.969, *p* = 1.56x10^-6^, 95% CI [.087, .202]). Contrary to expectation, an interaction emerged showing higher neighborhood educational opportunity was associated with lower gestational age acceleration at birth for neonates with mothers living in moderate to high HI (*B* = -.048, *t* = -2.08, *p* = .03, 95% CI [-.092, -.002]). Female neonates showed higher gestational age acceleration at birth compared to males. However, within males, being born into neighborhoods with higher social and economic opportunity was associated with higher gestational age acceleration.

**Conclusion:**

Prenatal HI and neighborhood qualities may affect gestational age acceleration at birth. Therefore, policy makers should consider neighborhood qualities as one opportunity to mitigate prenatal developmental effects of HI.

## Introduction

More than 43 million people live in low income households in the United States [1]. Children raised in a low income environment have been associated with a spectrum of negative health outcomes such as poor academic performance, fewer years of school completion [2], behavioral problems, and increased neonatal mortality rates [3, 4]. Effects of low household income (HI) have been found beginning in utero suggesting lower birth rate [5], increased risk for preterm birth [6], altered cortisol regulation [7], and through to adulthood when adult working memory capacity has been found to be negatively impacted [8]. Moreover, the consequences of low HI on fetal development are indicative of biological embedding that can perpetuate a cycle of generational income patterns making it difficult for subsequent generations to achieve better economic status [9].

Neighborhood factors can promote healthy child development such as higher IQ [10], overall cognitive processes, and healthy psychophysiology [11]. The impact of protective factors (such as access to community resources) on ameliorating the effects of childhood low HI are less clear. Roubinov and colleagues [12] found that neighborhood opportunities may protect against some of the stress and health consequences of living in a low income household. Children living in neighborhoods with higher opportunities did not show any association between HI and cortisol or physical health outcomes [13]. Therefore, neighborhood opportunities may prevent or reduce the biological consequences of stress associated with a low HI. Yet, it is unknown if the potential buffering effects of neighborhood opportunities could affect HI-linked outcomes in prenatal development. The mechanisms of how HI may affect prenatal development are not well understood, or how maternal exposure to a beneficial factor, such as neighborhood opportunities, may ameliorate the effects of low HI on prenatal development. Maternal exposure to neighborhood opportunities may mitigate the effects of low HI on the developing fetus.

Studies have found associations between childhood low HI and DNA methylation in adulthood, highlighting the potential long-term effects of low income exposure [8, 14]. Associations between DNA methylation and HI are evident even at birth, suggesting that prenatal exposure to low HI can affect DNA methylation patterns during fetal development [7, 15–18]. Changes in DNA methylation may be a mechanism underlying biological embedding of early-life exposure to low HI [14, 19]. Therefore, DNA methylation patterns established during childhood may create a trajectory of development that, when influenced by HI, could increase risk for low income-related outcomes in adulthood, including diminished negative emotion regulation [20] and higher allostatic load [21].

We have previously developed a DNA methylation-based biomarker of gestational age [22], DNA methylation gestational age (DNAm GA), that may provide novel insight into the effects of maternal income on development early in life. DNAm GA is a polyepigenetic score based on 148 CpG sites across the genome [22]. DNAm GA can be used to calculate a metric of developmental maturity, termed gestational age acceleration, which is the residual between DNAm GA and clinically estimated gestational age. This biomarker may offer a new perspective on how prenatal exposure to HI affects neonatal development. We have previously demonstrated that developmental age is associated with maternal Medicaid status in the CANDLE cohort, an indicator of HI [22]. The benefit of using Medicaid status as an indicator of HI is it includes a large proportion of individuals living with lower income. However, there may be beneficial knowledge gained from exploring the spectrum of HI in terms of the effects on gestational age acceleration at birth. Furthermore, we wanted to provide a more refined assessment of the interplay between the presence and absence of different neighborhood opportunity types in relation to developmental maturity. Therefore, we included a Childhood Opportunity Index (COI) previously created to map opportunities by neighborhood that include sub-domains of health and environment, social and economic, and education [23] following the protocol described in earlier research [13].

Previous research has identified sex-specific associations between HI and varying developmental outcomes, such as chronic low income associating with poor cognitive developmental effects only in girls [24, 25]. Furthermore, developmental age differences based on neonatal sex have been previously demonstrated [26–28] and these differences may be due to neuroendocrine variations between male and female fetus development [29, 30]. Such biological differences may also result in sex-specific patterns of DNA methylation [31] that could result in different sensitivities to the social environment [29]. Based on the distinct biological changes by sex, prior sex-specific findings, and previous associations of developmental age with sex, it is vital to consider sex-specific associations between HI and DNAm. This addresses a critical gap in the literature as sex-specific differences are often understudied and may have significant impacts on the interpretation of findings [32, 33]. Considering sex-specific associations will help inform professionals engaged in areas of child development about unique needs between male and female children in relation to prenatal HI. We sought to identify associations between mother’s income, their neighborhood opportunities, newborns’ gestational age acceleration, and the potential for newborn sex to moderate associations by leveraging a subset of data from the CANDLE cohort [34–36]. This study is not published or considered elsewhere.

## Methods

### Cohort description

This study meets ethical principles of the Helsinki Declaration, protection of human subjects outlined in the Belmont Report, was approved by the CANDLE ethical oversight committee, which is the research group IRB, and was exempt from IRB review at Emory and Syracuse Universities given the de-identified secondary data analysis. Data used for this study are available through CANDLE and collaboration application information can be found on their website (https://candlestudy.uthsc.edu/collaboration/). The study data are from the CANDLE cohort (Conditions Affecting Neurocognitive Development and Learning in Early Childhood) who participated in the original data collection from and were accessed in June 2018. Details of this cohort are previously described [36]. Briefly, this cohort consists of 198 mother-neonate pairs recruited from prenatal clinics in western Tennessee. Selected participants were of ages 16–40 years, having a singleton pregnancy and low-risk pregnancy status.

### Household income

Household income (HI) was self-reported by mother participants and adjusted for the number of household members. Household income was divided by the square root of the number of people in the family and the correct income adjustment was noted for the household. Income was reported by mothers and treated as continuous and categorical when divided into moderate-high- and low-income groups. Categorical HI was used to further discern the effects of income when appropriate. HI was reported in increments of 5,000 to 10,000 with a range of 0 to 75,000 or greater. The majority of participating mothers reported income, after adjustment, within $20,000 and $44,999 annually. Low household income was determined to be adjusted HI ≤ $24,999 according to federal poverty guidelines 2024 and the income categories closest to the determined value ($25,820) for a family of three [37].

### Neighborhood opportunities

Neighborhood opportunities, as measured by the Childhood Opportunity Index (COI), were compiled following previously developed protocol using publicly available data from nationally representative surveys [23]. The COI development was outlined by Acevedo-Garcia and colleagues [23]. The Child Opportunity Index and its three opportunity domains are calculated for all census tracts in the 100 largest US urban regions. About 4,000 people and 1,600 homes live in each census tract. A metropolitan area includes a center urban district with over 50,000 people and surrounding counties that are socially and economically integrated. Research and conceptual frameworks informed on how neighborhoods affect child development. Z-scores compared a neighborhood’s opportunity indicators to those of other neighborhoods in the region. This method measures neighborhood data by proximity to regional averages. The three domains’ opportunity indices are calculated by averaging each indication’s z-scores. Finally, the domain indices are averaged to calculate the opportunity index [38].

The COI was computed into an overall summary score and sub-domains of education, social and economic, and health and environment opportunities, each treated as continuous variables. The COI sub- domains include data that can be used to inform on opportunities available for children and parents [23]. The health and environment domain includes measurements such as proximity to parks and healthcare facilities, nearby toxic waste, and the housing vacancy rate. The social and economic domain includes measures of poverty, unemployment, proximity to available work, and public assistance. The education domain consists of measures of local area students’ math and reading proficiency, proximity to quality early-childhood learning centers, high school graduation rates, and adult education attainment. Seventeen participants had random missing values among income status, COI, and race variables so we employed pairwise deletion to address the missing data.

### Covariates

Neonate sex, race, and gestational age were derived from a neonate summary form completed after birth. Sex was coded as a binary variable (male = 0, female = 1). Race was coded with categories including African American, Caucasian, Asian, and “other”. While the race categories were not exhaustive and did not include an ethnicity option for Hispanic or Latino, this is due to the predominant African American and Caucasian population distribution within the geographic area of this study sample from the west Tennessee region. Only one participant was identified as Asian, and four identified as “other”. Four percent of participants were missing race data and the remaining 93.5% of participants were identified as African American or Caucasian. Gestational age was treated as a continuous variable with a range of 30–44 weeks estimated gestation from the neonate summary form completed by the mother’s medical care provider.

### Biological sample collection and DNA extraction

The hospital staff obtained umbilical cord blood using conventional protocols. The entire blood sample was then utilized to extract and evaluate DNA methylation and gene expression. The DNA extraction was conducted by CANDLE researchers at the Department of Health and Science of the University of Tennessee in Memphis using the Wizard Genomic DNA purification kit (Promega Corp.). The DNA was bisulphite-converted using the EZ-96 DNA Methylation kit from Zymo Research. Samples were processed on the HumanMethylation27 BeadChip according to manufacturer’s specifications (Illumina Inc.). An initial data quality assessment was performed using the R package CpGassoc [39]. CpG sites exhibiting low signal or containing missing data in over 5% of samples were excluded, as were samples with missing data for over 5% of CpG sites, no samples or CpG sites failed this initial QC. Subsequently, cross-reactive, and polymorphic CpGs were deleted. The beta values (β) for each CpG site were computed by dividing the methylation signal (M) by the sum of the methylated and unmethylated signals (M+U): β = M/M+U. DNA methylation QC was conducted as previously described [22]. Samples were checked for sex match discordance and no samples failed. The ComBat method was employed to mitigate the influence of chip and position effects that could potentially distort the results [40]. The revised dataset was utilized to estimate gestational age acceleration as previously described by Knight and colleagues (2016) [22]. A Cross reference check of the gestational age acceleration (GAA) CpG sites and the Illumina HumanMethylation27K annotation file revealed that all 148 CpG sites used to estimate GAA were included in the HumanMethylation27K data for the neonates.

### Statistical analysis

Gestational age acceleration was regressed on prenatal household income (HI) and maternal exposure to COI in separate and combined linear models while controlling for neonate sex, race, and cellular composition estimated from methylation data [41]. A second analysis regressed interaction variables containing (1) neonate sex and HI, and (2) neonate sex and COI on gestational age acceleration to investigate moderating effects. All interactions tested used a significance cutoff of p < .05. A sex-stratified analysis was conducted to extrapolate meaningful details in the sex-specific associations while controlling for the appropriate covariates. All analyses were conducted in R version 1.1.383 [42].

## Results

### Variable descriptive statistics

**Fig 1:**
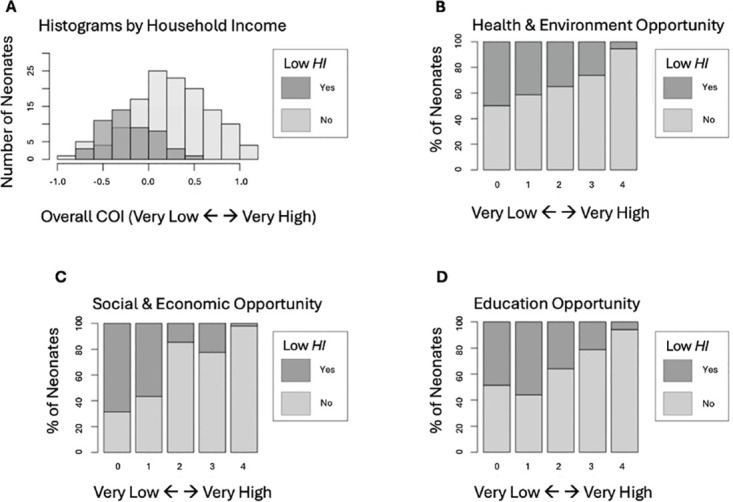
Histograms by household income status. **(A)** Mothers living in moderate to high household income showed greater access to neighborhood opportunities overall (B = .065, t = 9.166, p < 2x10^-16^), **(B)** health and environment (B = .123, *t* = 4.513, *p* = 1.08x10^-5^), **(C)** social and economic (B = .217, *t* = 9.507, *p* < 2x10^-16^), and **(D)** education opportunities (B = .159, *t* = 6.565, *p* = 4.25x10^-10^).

**Fig 2:**
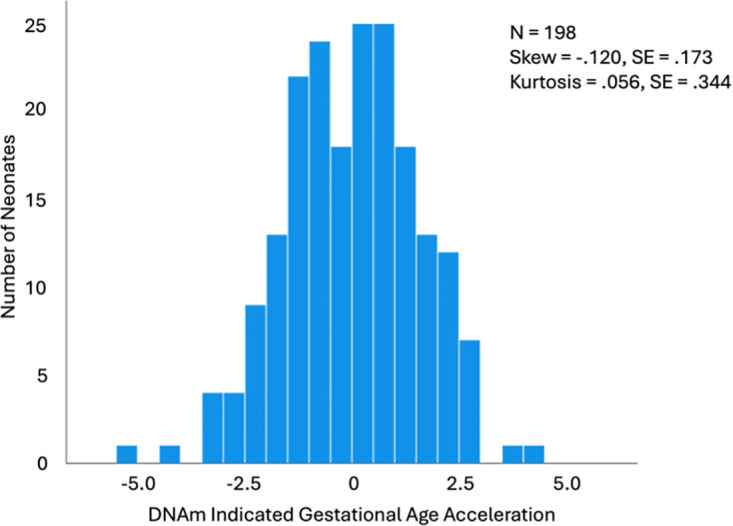
Histogram of neonate gestational age acceleration. Neonate gestational age acceleration is normally distributed (*M* = 0, *SD* = 1.54, *Min* = -5.01, *Max* = 4.36) with an age above “0” indicative of greater physical development for the gestational age at birth.

The majority of neonates in this subset (N = 198) were male (52%) and mostly African American (n = 102). Caucasian accounted for the next largest group of neonates (n = 91), and some neonates identified as “other” (n = 4), or Asian (n = 1) with 4% of participants missing race data. As the HI increased for mothers so did the COI overall (B = .065, *t* = 9.166, *p* < 2x10^-16^, 95% CI [.053, .082]), health and environment (B = .123, *t* = 4.513, *p* = 1.08x10^-5^, 95% CI [.068, .182]), education (B = .159, *t* = 6.565, *p* = 4.25x10^-10^ 95% CI [.121, .219]), and social and economic (B = .217, *t* = 9.507, *p* < 2x10^-16^, 95% CI [.171, .264]) opportunities (Fig 1A–1D). Though the subdomains are moderately correlated (r’s = .50 to .64; p< .05), they each provide independent information (variance inflation factors < 5). Gestational age acceleration was normally distributed as shown in Fig 2 (*M* = 0, *SD* = 1.54, *Min* = -5.01, *Max* = 4.36).

### Household income, neighborhood opportunity, and gestational age acceleration

**Fig 3:**
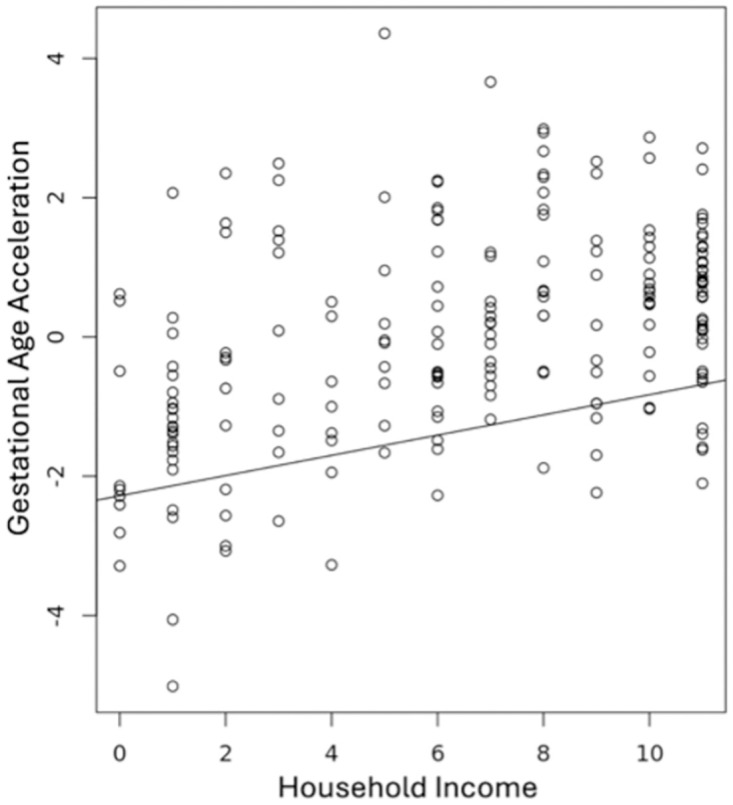
Household income association with gestational age acceleration. Prenatal household income (x-axis) positively associated with gestational age acceleration (y-axis) after controlling for neonatal sex, race, and cell composition (B = .145, *t* = 4.969, *p* = 1.56x10^-6^).

In the full sample, gestational age acceleration was positively associated with HI showing increased gestational age acceleration with higher HI and decreased gestational age acceleration with lower HI after controlling for sex, race and cell type (B = .145, *t* = 4.969, *p* = 1.56x10^-6^, 95% CI [.087, .202], Fig 3). Due to small group sizes, we collapsed child race into Caucasian and non-Caucasian neonates to investigate within-race HI associations. Only 2% of Caucasian neonates had mothers living in a low HI compared to 48% of non-Caucasian neonates, and as a result we were not adequately powered to examine within-race HI associations with gestational age acceleration in Caucasian neonates. However, non-Caucasian neonates showed the same positive association between HI and gestational age acceleration as found in the full cohort (B = .134, *t* = 3.167, *p* = .002, 95% CI [.050, .218]).

We next examined whether prenatal COI was associated with gestational age acceleration at birth. Overall COI did not associate with gestational age acceleration (p > .05).

**Fig 4:**
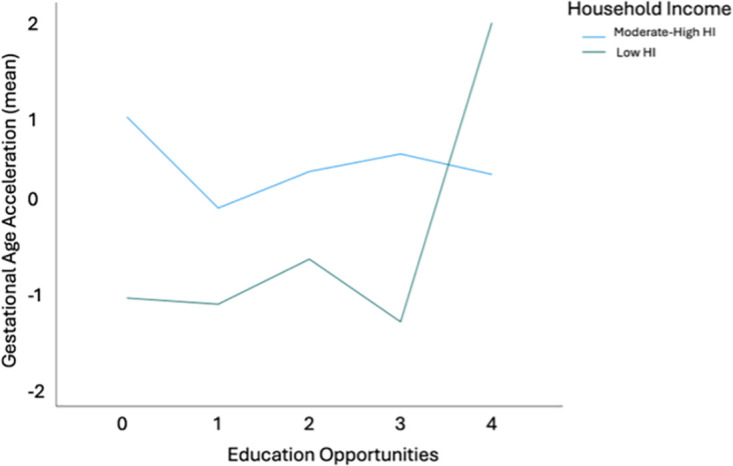
Gestational age acceleration by education opportunity in neonates exposed to mod-high household income. Neonates born into neighborhoods with more education opportunity (x-axis) have lower gestational age acceleration (y-axis) (B = -.048, *t* = -2.08, *p* = .03). Gestational age acceleration is graphed in mean values.

Analysis of the subdomains of COI showed education (p > .05), health and environment opportunities (p > .05), and social and economic opportunities (p > .05) did not associate with gestational age acceleration. However, previous findings revealed higher cortisol levels with lower COI in children living in low HI, but lower cortisol levels with lower COI in children living in high HI [13]. Therefore, we stratified the sample by low/mod-high HI status to assess for association differences by HI. COI did not associate with gestational age acceleration in neonates prenatally exposed to a low HI (Overall COI, *p* > .05; Education, *p* > .05; Health and Environment, *p* > .05; Social and Economic, *p* > .05). However, mothers living in mod-high HI with higher education opportunities had neonates with lower gestational age acceleration at birth compared to peers living in low HI with the highest education opportunities (B = -.048, *t* = -2.08, *p* = .03, 95% CI [-.092, -.002], Fig 4). Overall COI (*p* > .05), health and environment (*p* > .05), and social and economic opportunities (*p* > .05) did not associate with gestational age acceleration in neonates born into mod-high HI.

### Neonatal sex as a moderator of COI and household income associations with developmental age

**Fig 5:**
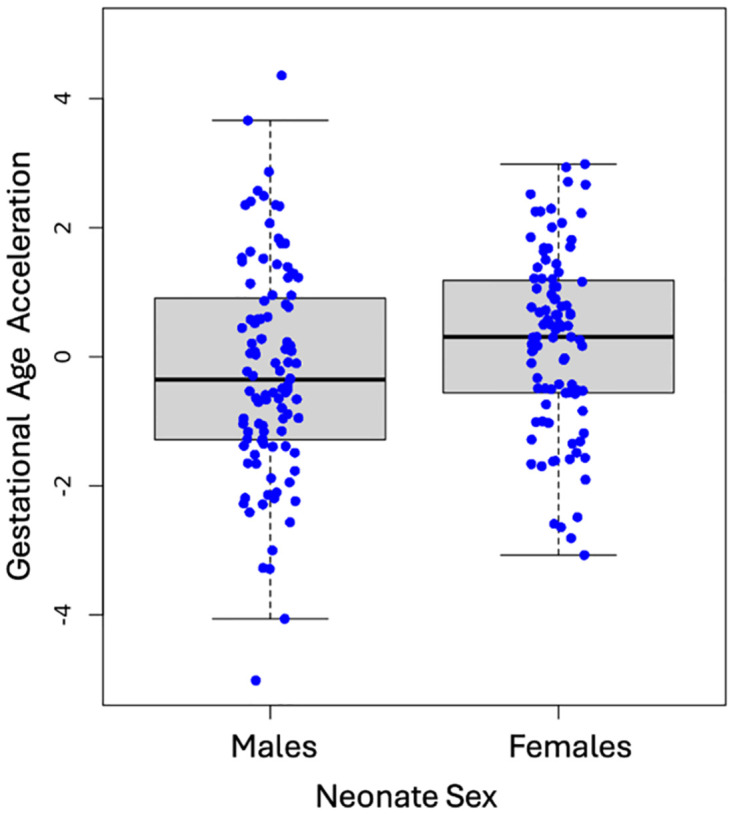
Neonatal sex association with gestational age acceleration. Female neonates (x- axis) have higher gestational age acceleration (y-axis) compared to male neonates at birth (B = .794, *t* = 3.415, *p* = .0007).

We analyzed the phenotypes for differences by neonatal sex controlling for child race. Overall COI (B = .166, *t* = 2.75, *p* = .006, 95% CI [.046, .285]), and social and economic opportunity (B = .602, t = 3.137, p = .002, 95% CI [.223, .980]) varied between males and females (Table 1). We further assessed developmental age for sex differences and found female neonates showed higher gestational age acceleration at birth compared to male neonates after controlling for race and cell composition (B = .794, *t* = 3.415, *p* = .0007, 95% CI [.335, 1.253], Fig 5). We evaluated the interaction of neonatal sex and HI on gestational age acceleration and found no moderating association (*p* > .05). The interaction analysis for neonate sex and COI overall also proved no association for moderating effects on gestational age acceleration (*p* > .05). The subdomains of COI also did not show interactions with neonate sex on gestational age acceleration (health and environment: *p* > .05; social and economic: *p* > .05; education: *p* > .05). Given that interaction models can require large statistical power and that our cohort neonate gestational age acceleration varied by sex, we sex-stratified the sample to explore the potential for smaller associations that could inform the complexity of the environmental influence.

**Table 1:** Demographic, risk, and protective factors by neonate sex.

*Variable*	*Full Sample*	*Males N (%)*	*Females N (%)*
Neonate Sex	198	103 (52%)	95(48%)
Non-Caucasian	107	60 (30%)	47 (24%)
Household Income (Mean±SD)	22,739±17,820	21,049±18,648	24,412±16890
COI: Overall (Mean±SD)	.09±.45	.02±.42	.17±.47
COI: Health & Environment (Mean±SD)	2.24±1.45	2.05±1.46	2.45±1.41
COI: Social & Economic (Mean±SD)	2.17±1.41	1.91±1.48	2.44±1.29
COI: Education (Mean±SD)	2.3±1.37	2.25±1.29	2.35±1.45

**Fig 6:**
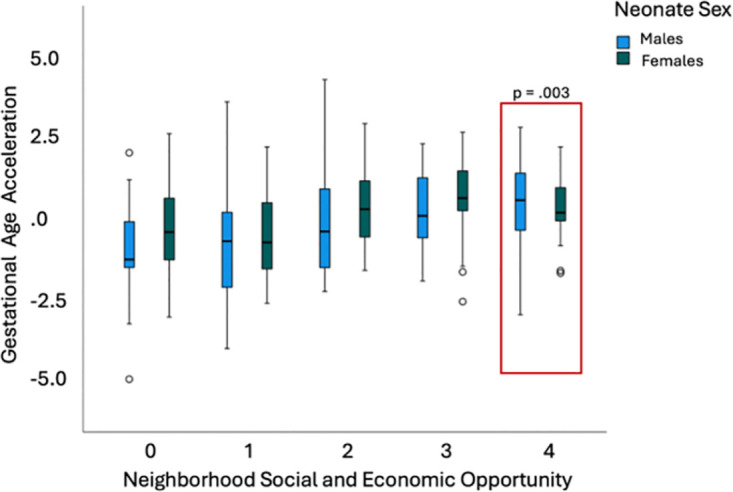
Neonatal sex moderates association among social & economic opportunity and gestational age acceleration. Males exhibit higher gestational age acceleration with highest neighborhood social and economic opportunity compared to females (B = .335, *t* = 3.096, *p* = .003).

COI did not associate with gestational age acceleration in female neonates: overall COI (*p* > .05), health and environment (*p* > .05), education (*p* > .05), and social and economic opportunities (*p* > .05). Likewise, overall COI did not associate with gestational age acceleration in male neonates (*p* > .05), health and environment (*p* > .05), or education (*p* > .05). However, males did exhibit higher gestational age acceleration with higher neighborhood social and economic opportunity (B = .335, *t* = 3.096, *p* = .003, 95% CI [.120, .550], Fig 6). A summary of HI associations is included in Table 2, and neonate sex associations are summarized in Table 3.

**Table 2:** Household income associations.

*Model*	*Coeff*. *(B)*	*t-statistic*	*p-value*	*95% CI*
HI→COI	.065	9.166	< 2x10^-16^	[.053, .082]
HI→Health & Environment	.123	4.513	1.08x10^-5^	[.068, .182]
HI→Education	.159	6.565	4.25x10^-10^	[.121, .219]
HI→Social & Economic	.217	9.507	< 2x10^-16^	[.171, .264]
HI→Gestational Age Acceleration	.145	4.969	1.56x10^-6^	[.087, .202]
Non-Caucasian HI→Gestational Age Acceleration	.134	3.167	.002	[.050, .218]
HI*Education→Gestational Age Acceleration	-.048	-2.08	.03	[-.092, -.002]

**Table 3:** Neonate sex associations.

*Model*	*Coeff*. *(B)*	*t-statistic*	*p-value*	*95% CI*
Sex→COI	.166	2.75	.006	[.046, .285]
Sex→Social & Economic	.602	3.137	.002	[.223, .980]
Sex→Gestational Age Acceleration	.794	3.415	.0007	[.335, 1.253]
**Males subset** Social & Economic→ Gestational Age Acceleration	.335	3.096	.003	[.120, .550]

## Discussion

This is the first study, to our knowledge, to investigate the direct and interaction effects of neighborhood opportunity (measured with the childhood opportunity index), household income, and neonate sex on gestational age acceleration. A recent study examined the childhood opportunity index and age acceleration in children as young as 6 years and found a positive correlation across several age acceleration clocks [43]. It is possible that our null findings for direct effects of COI were due to the indirect exposure through the mother during pregnancy.

In this study, we extended and supported our previous findings of lower socioeconomic status associations with lower gestational age acceleration at birth [44] with a different indicator of SES, specifically household income (HI). Lower gestational age acceleration was associated with prenatal lower household income. Previous research has shown a positive association between children and teenagers aged 8–18 years living in impoverished homes and areas and the rate at which they age measured with age acceleration residuals [45]. The results of our study contradict the expected direction of the relationship, yet the negative effects on biology are comparable in terms of risk. A newborn with lower gestational age acceleration has less physical development compared to their peers, which consequently puts them at a higher risk for possible health problems. Children who experience accelerated aging would likewise face heightened health risks.

Our direct effects of sex findings are consistent with previous research suggesting male neonates have lower gestational age acceleration compared to female neonates at birth [46, 47] but are in contrast to prior research on twins showing young adult males have increased age acceleration compared to female peers [48]. However, higher gestational age acceleration was found in male neonates with mothers who reported greater social and economic opportunity in the neighborhood. This could be due to variation in the neuroendocrine milieu during prenatal development, but additional research is needed to discern specific mechanisms and pathways. Future studies could investigate race as a potential confounder and assess the influence of different neighborhood opportunities within the context of race and sex-specific associations with gestational age acceleration.

We did detect lower gestational age acceleration in newborns that did not experience prenatal exposure to low household income and were born in neighborhoods with more education opportunities. It is possible that the limited neighborhood opportunities available to our participants living in a lower household income likely reduced our power to detect more complex relationships between maternal household income, neighborhood opportunities, and neonate gestational age acceleration. Replication of this study in a larger sample may identify neighborhood opportunities that interact with household income to provide protective effects for the developing fetus. Furthermore, the difference in gestational age acceleration between male and female neonates supports the need to assess results for varying effects on males and females. In time, the implications for practice could include improved health and development of intervention and prevention strategies for children according to their household income.

### Limitations

There are some limitations to this study. First, the sample is from one urban community in western Tennessee and therefore generalizability may be limited. Furthermore, this study had a moderate sample size that was reduced in stratified sample analyses. Although sex-stratified analyses provide more meaningful information for interpretation, the reduced sample size also reduces statistical power. Additionally, fewer individuals living in a low household income resided in the highest-opportunity neighborhoods whereas those living with more economic means lived in neighborhoods across the full spectrum of opportunity (see Fig 1). Therefore, our ability to optimally test for interactions between COI and household income in association with gestational age acceleration was limited. Lastly, an alternative gestational age acceleration predictor [49] that could have been used to compare results is incompatible with the DNA methylation array used for this study. Future research on gestational age acceleration would benefit from including a more diverse racial and household income sample to discern effects associated with race and household income separately. Despite these limitations, this study provides important initial examinations of unstudied gestational age acceleration in newborns associated with neighborhood characteristics and prenatal household income which can provide valuable insight for prevention planning to promote healthy prenatal development.

### Future implications

This study shows household income and neighborhood characteristics can have intergenerational effects on neonate gestational age acceleration at birth. Furthermore, social and economic opportunity mitigated the lower gestational age acceleration in male neonates. Improving our understanding of how we differ between the sexes in our adaptation to the environment will aid our endeavors to improve quality of life for those living with disadvantage and adversity.
